# Bioremediation of Crude Oil Contaminated Desert Soil: Effect of Biostimulation, Bioaugmentation and Bioavailability in Biopile Treatment Systems

**DOI:** 10.3390/ijerph13020219

**Published:** 2016-02-16

**Authors:** Farid Benyahia, Ahmed Shams Embaby

**Affiliations:** 1 Department of Chemical Engineering, Qatar University, Doha 2713, Qatar; 2 Chemical Engineering Department, College of Engineering, United Arab Emirates University, Al Ain 15551, United Arab Emirates; ahmad.embaby@gmail.com; 3 Worley-Parsons Environment, Kuwait City 9912, Kuwait

**Keywords:** desert soil bioremediation, biostimulation, bioaugmentation, bioavailability, bioaccessibility

## Abstract

This work was aimed at evaluating the relative merits of bioaugmentation, biostimulation and surfactant-enhanced bioavailability of a desert soil contaminated by crude oil through biopile treatment. The results show that the desert soil required bioaugmentation and biostimulation for bioremediation of crude oil. The bioaugmented biopile system led to a total petroleum hydrocarbon (TPH) reduction of 77% over 156 days while the system with polyoxyethylene (20) sorbitan monooleate (Tween 80) gave a 56% decrease in TPH. The biostimulated system with indigenous micro-organisms gave 23% reduction in TPH. The control system gave 4% TPH reduction. The addition of Tween 80 led to a respiration rate that peaked in 48 days compared to 88 days for the bioaugmented system and respiration declined rapidly due to nitrogen depletion. The residual hydrocarbon in the biopile systems studied contained polyaromatics (PAH) in quantities that may be considered as hazardous. Nitrogen was found to be a limiting nutrient in desert soil bioremediation.

## 1. Introduction

Bioremediation of soils contaminated by hydrocarbons is an established method these days and has been put in practice in several ways such as “*in-situ*” or “*ex-situ*” technologies [1,2,3,4,5]. However, the effectiveness of soil bioremediation, both technical and economic, has been much debated and is still the subject of numerous research investigations. This stems from the fact that soils, soils constituents and contaminants vary to a great extent, thus making interactions and dependencies between these extremely complicated. Some of these intricacies have been reviewed recently [4]. Micro-organisms being at the forefront of the contaminated soils treatment have also been thoroughly investigated [3,5,6,7,8]. For instance a recent paper by Roy *et al.* [3] indicated that up to 39 native crude oil degrading bacteria can be found at contaminated sites and that these were dominated by the *Pseudomonas* genus. The work of Suja *et al.* [7] also involved *Pseudomonas* bacteria amongst other strains. In virtually all cases of field tests of bioremediation in the literature either native or naturally occurring bacteria added in the bioremediation treatment have been used. The idea of using “genetically engineered” micro-organisms still face regulatory hurdles because of the unknown consequences that may ensue the release in nature of such manipulated micro-organisms [4]. 

The major factors affecting the effectiveness of hydrocarbon contaminated soils typically include the presence of biomass in sufficient quantity, adequate nutrients and “optimum” conditions such as moisture, pH and temperature [9]. Where soils are considered “poor”, a number of measures need to be taken such as amendments and addition of biomass (bioaugmentation) and nutrients (biostimulation). The traditional recommended ratio between carbon (C) and other critical nutrients such as nitrogen (N), phosphorus (P) and potassium (K) often denoted collectively as CNPK, or leaving out potassium as CNP appears to vary according to the literature [10,11,12]. In addition, amongst the important nutrients, nitrogen was reported to be one of the significant limiting nutrient when bioremediation action is monitored [10,12,13,14]. Xu and Lu [15] compared the efficiency of biostimulation and bioaugmentation treatments of crude oil contaminated soil using peanut hull powder as biomass immobilization medium. They reported a biodegradation enhancement ranging from 26% to 61% over a period of 12 week treatment. They also observed a biodegradation improvement when peanut hull powder was added as a bulking agent only and attributed the enhanced treatment to improved nutrients, water and oxygen transport in the “clay-loam” soil being treated.

Given the complex interactions between soil biomass, hydrocarbon contaminants and other nutrients, there has been a great deal of debate about the so-called “bioavailability” of nutrients. This stems from the fact that generally most hydrocarbons are considered as hydrophobic and may therefore not be supplied adequately to micro-organisms compared to water soluble nutrients. Hence, additives that facilitate nutrient transport within the soil matrix during bioremediation have been tested. These were invariably surfactants, mainly nonionic and sometimes bioemulsifiers [16,17]. The definition and scope of “bioavailability” has been discussed by Joop Harmen [18] and elaborated further by Semple *et al.* [19] with the introduction of “bioaccessibility” as a useful term in the context of soil bioremediation and interaction between micro-organisms and nutrients scattered in the soil matrix.

Successful outcomes of bioremediation of hydrocarbon contaminated soils have generally been measured through reduction in total petroleum hydrocarbon (TPH) content. This bulk parameter does not really discriminate between the organic species in the petroleum fractions and there has been some concerns about the toxicity of residual hydrocarbons. In that respect, polyaromatic hydrocarbons (PAH) were given particular attention [20,21,22]. Soil respiration has also been widely reported as a measure of microbial activity in hydrocarbon biodegradation [23].

Desert soils are generally considered poor in terms of nutrients and micro-organisms due to the harsh climate and very low rainfalls. When such soils become contaminated by hydrocarbon spills, remediation is often problematic and most oil industries resort to landfarming practices. Very little detailed information is currently available about effective mitigation of crude oil contaminated desert soils in the Arabian Peninsula. This work addresses important issues related to bioremediation of a specific desert soil close to an important oil installation in the United Arab Emirates. A biopile system has been employed in this study since landfarming is known to transfer a significant part of the crude oil spill to the atmosphere through evaporation in the early part of the spill and soil ploughing. A major objective of this work is to evaluate the relative merit of biostimulation, bioaugmentation and bioavailability in the biopile treatment process of an artificially contaminated desert soil and draw conclusions aimed at improving future treatment processes of crude oil contaminated soils in the region.

## 2. Experimental Section

### 2.1. Soils and Biopile Formulations

**Figure A1:**
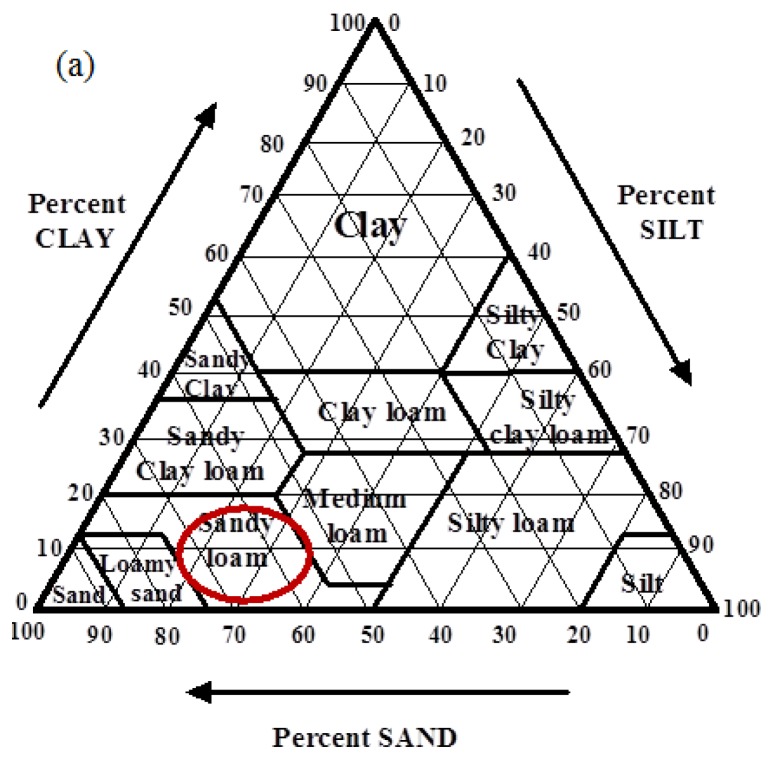
Soil texture from Sahel oil field (Abu Dhabi, UAE).

In this work, five batches of soils were considered; four of which were prepared from clean soil for bioremediation experiments and one was kept clean, used to determine texture and other properties. The clean soil was collected from Sahel oil field in Abu Dhabi (United Arab Emirates) at depth of around 60 cm from the surface. The texture was determined gravimetrically as “sandy loam” after sieving according to American Society for Testing and Materials (ASTM) method for soil classification and the soil texture triangle depicted in Figure A1 in the Appendix. The clean soil physical properties determined experimentally are conveniently summarized in Table A1 in the Appendix. It can be seen that the clean Sahel oil field soil is “sandy loam”, slightly alkaline and with an absorption capacity of 23% wt/wt and 17% wt/wt for water (field capacity) and crude oil respectively. Starting from clean soils, four soil formulations were prepared for biopiles, representing artificially contaminated soil by addition of crude oil with characteristics shown in Table 1 and other additives according to the objectives of this work, namely to study the effect of biostimulation, bioaugmentation and bioavailability in biopile treatment of crude oil contaminated desert soils. 

**Table 1:** Biopile systems formulation.

Biopile tag	Clean Soil (g)	Crude oil (g)	Urea-N (g)	K_2_HPO_4_-P (g)	K_2_HPO_4_-K (g)	Amnite P300 (g)	Tween 80 ** (g)
Bio_Cont	1850	277.5	--	--	--	--	--
Bio_Stim	1850	277.5	23.8–11.1	9.74–2.2	9.74–5.5	--	--
Bio_Aug	1850	277.5	23.8–11.1	9.74–2.2	9.74–5.5	3%–55.5	--
Bio_Avail	1850	277.5	23.8–11.1	9.74–2.2	9.74–5.5	3%–55.5	157.25 *

* Amount is 10×CMC. CMC is critical micelle concentration; ** IUPAC name: polyoxyethylene (20) sorbitan monooleate.

The formulated biopile systems were tagged as “Bio_Cont” for the system in which only crude oil was added to clean soil and hence will serve as a form of control system with indigenous micro-organisms, “Bio_Stim” for the system in which crude oil and nutrients were added and will represent bio-stimulated indigenous micro-organisms, “Bio_Aug” for the system in which crude oil, nutrient and Amnite P-300 is added to represent bio-augmented indigenous micro-organisms and finally “Bio_Avail” for the system in which crude oil, nutrients, Amnite P300 and the surfactant polyoxyethylene (20) sorbitan monooleate (Tween 80) were added to represent a bioaugmented system with an nonionic surfactant to enhance nutrient bioavailability. The nitrogen, phosphorus, potassium (NPK) nutrients amounts are displayed in Table 1 as a second number following the total amount of the salt from which they were derived. The nonionic surfactant Tween 80 was added as ten times the critical micelle concentration. Amnite P300 [24] is a commercially available bacterial product (Cleveland Biotech, Stockton, UK) consisting of a consortium of 10 strains belonging predominantly to the *Pseudomonas* genera. The total viable count of this product is not less than 5 × 10^8^ CFU (Cleveland Biotech). The bacteria are immobilized in a cereal carrier.

After crude oil and formulation additives were put in the clean soils, a thorough blending was performed before loading the contaminated soils into their respective biopile systems described in the next section.

### 2.2. Biopile Set-Ups

**Figure A2:**
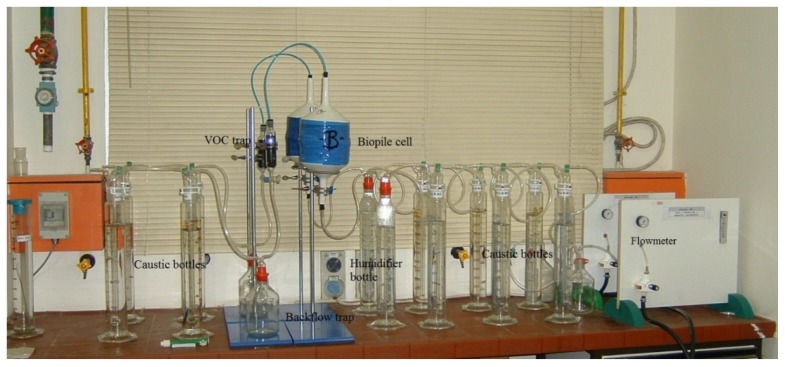
Biopile systems used in this work.

The bioremediation treatment systems employed in this work are based on self contained biopile conical enclosures made of ceramic material. The bottom ceramic cone has a porous base on which a geotextile cloth was placed to allow aeration and prevent fine soil material dropping. The top ceramic inverted cone served to channel the gasses (respiration product, volatile hydrocarbons, residual air) to a VOC trap (small granular activated carbon bed) and a series of gas washing bottles containing NaOH solution aimed at collecting atmospheric and respiration carbon dioxide. These bottles had sampling ports for daily monitoring of respiration rates. The biopile air was supplied from a compressor through a pressure regulator and a needle valve flowmeter at 0.5 L/min to ensure adequate oxygen is provided. After metering, air was washed in a series of caustic traps (gas washing bottles) to remove atmospheric carbon dioxide then air is washed in distilled water (humidifier bottle) to remove any entrained caustic and also to transport humidity to the biopile soil. The biopile effluent stream is washed in a series of caustic solution gas washing bottles for the determination of respiration CO_2_. There were four separate biopile trains containing the formulations presented in Table 1. Figure A2 in the Appendix depicts an image of two such biopile systems.

### 2.3. Analytical Methods

#### 2.3.1. TPH and TKN

The hydrocarbon content of the biopile systems was determined before and after treatment by measuring the total petroleum hydrocarbon (TPH) using an “in-house” gravimetric and Fourier Transform Infrared spectroscopy (FTIR) method with acid treatment (typically pH around 2) and soxhlet extraction followed by rotary evaporation of solvent). The procedure outline is as follows: Homogenized soil (5 g, dried at room temperature) was accurately weighed and transferred to a glass mortar. The sample was acidified to pH 2 with approximately 0.1 mL of concentrated HCl (10.1). Prepared MgSO_4_ (5.0 g) was added to the acidified sample until it was free flowing, mixed well, and left for about 15–30 min at room temperature. The material was ground into a fine powder. A clean, dry (with automated Soxhlet) aluminum cup or round bottom flask was weighed and the weight recorded. The powder was quantitatively transferred into the extraction thimble. The mortar and pestle were washed with hexane (about 10 mL) and the contents transferred. The thimble was placeced in a Soxhlet apparatus and extracted using *n*-hexane (about 70 mL) for 6 h (automated Soxhlet system) and 16 h (manual Soxhlet). The solvent was removed, the sampled cooled and the extraction cup which contained the residue (TPH) removed. The cup was dried in a desiccator for 15 min. and weighed again. The TPH (mg/kg) was calculated from the weight of residue and the sample. When the TPH was less than 1000 mg/kg, it was determined by FTIR. The residue in the cup was dissolved in trichlorotrifluoroethane, and quantitatively transferred into a 25 mL volumetric flask and made up to volume. TPH was calculated as mg/kg of sample. The total Kjeldahl nitrogen (TKN) was determined according to the US Environmental Protection Agency method 351.2.

#### 2.3.2. Determination of Respiration CO_2_

The biomass respiration rate was measured by means of an “in-house” developed titrimetric method. The respiration gas leaving the biopile cell was washed in a concentrated caustic solution (4 M) by means of two gas washing bottles in series fitted with gas diffusers to ensure complete dissolution of CO_2_. The number of bottles in series was determined experimentall to trap 100% of CO_2_ in the gas stream. Daily samples of the caustic solution were collected accurately for acid titration to determine CO_2_. The accurate titration was conducted automatically using a pH meter endowed DOSIMAT autotitrator (Metrohm, Herisau, Switzerland) and a titroprocessor to pinpoint the end points. To enhance the measurements, two color indicators were also employed (phenolphthalein and methyl orange).

### 2.4. Experimental Design

The four biopile treatment systems with formulations described in Table 1 were started simultaneously and the biopile respiration rates were monitored daily through titration as described above. The titration results were fed in a specially designed spreadsheet that calculates the CO_2_ evolved as daily respiration rate and as cumulative CO_2_ production. Care was taken to ensure the adequate liquid level and strength of the caustic solution which is replaced as and when required. Leak tests were performed frequently to ensure reliable results. Experiments were performed in a laboratory at constant ambient temperature of around 22 °C for an extended period and the daily spreadsheet calculations served as a guide on when it was time to stop runs and conduct the soil TPH/nutrient analysis after treatment.

## 3. Results and Discussion

The results of the biopile treatment processes will be presented and discussed in the following sub-sections by means of the patterns observed in respiration rates and CO_2_ evolved throughout the experiments, TPH removal efficiency, nutrient effect and PAH removal efficiency. 

### 3.1. CO_2_ Generation

**Figure 1:**
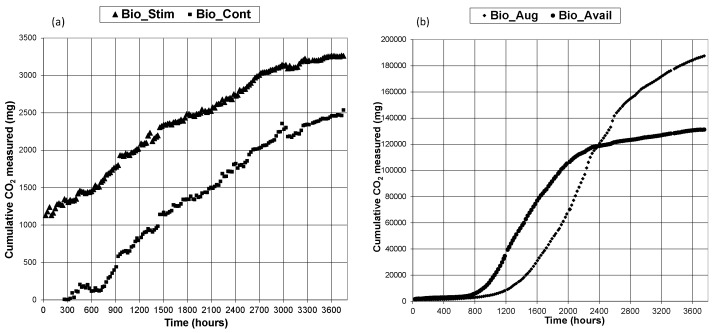
Combined cumulative CO_2_ generation (**a**) Bio_Cont and Bio_Stim (**b**) Bio_Aug and Bio_Avail.

**Figure 2:**
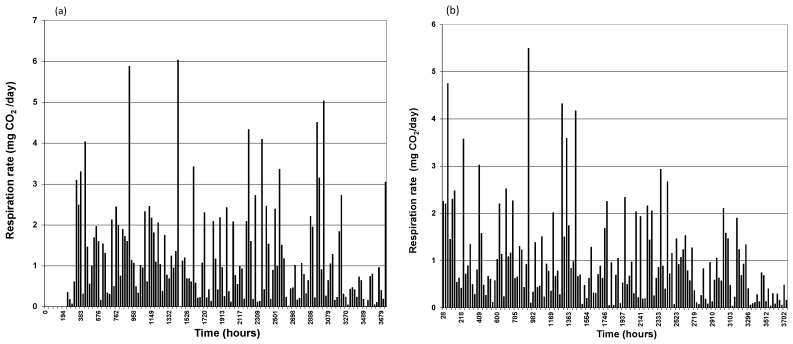
Instantaneous daily rates of CO_2_ generation for biopile (**a**) Bio_Cont, (**b**) Bio_Stim.

The cumulative CO_2_ evolved during biopile treatment of soils is presented in Figure 1a,b for each of the indigenous bacteria soil (Bio_Cont) that serves as control for comparison of performance, biostimulated indigenous bacteria (Bio_Stim), bioaugmented system (Bio_Aug) and bioaugmented system with enhanced bioavailability (Bio_Avail) respectively. The Bio_Cont system CO_2_ evolution in the graph in Figure 1a shows that there was a fairly long lag time of around 250 h before any measurable respiration CO_2_ was obtained. This can be considered as a reversible inhibition followed by an adaptation period for the indigenous biomass to start mineralizing hydrocarbons. On the other hand, the biostimulated indigenous biomass system started producing respiration CO_2_ within hours with no obvious indication of a long lag time, as shown in Figure 2a. 

**Figure 3:**
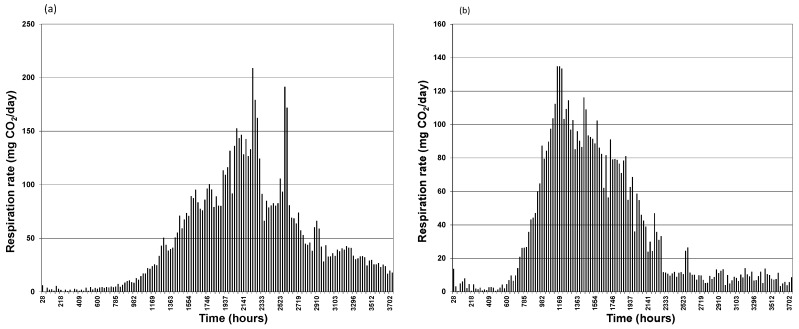
Instantaneous daily rates of CO_2_ generation (**a**) Bio_Aug, (**b**) Bio_Avail.

Likewise, the bioaugmented and bioavailable systems started producing respiration CO_2_ without significant lag time as shown in Figure 1b. The major difference between CO_2_ evolved with systems relying on indigenous biomass and bioaugmented systems is the amount of CO_2_ produced and the pattern of its production throughout the duration of the experiments. A close inspection of Figure 1a,b shows that there a significant order of magnitude difference in CO_2_ generation. Figure 1b represents the bioaugmented systems display typical “sigmoid” cumulative CO_2_ curves. This is not the case for indigenous bacteria systems, even with biostimulation. Sigmoid curves usually indicate different phases in the processes. This can be clearly seen in the graphs representing daily respiration rates. Figure 2a,b present daily respiration rates for the indigenous biomass and stimulated indigenous biomass systems indicate that there is no particular pattern in daily respiration which is a fair representation of the bacterial action on nutrient hydrocarbon mineralization. The respiration rates were generally low with occasional spikes. In contrast, bioaugmented systems daily respiration rates shown in Figure 3a,b for bioaugmented only and bioaugmented with enhanced bioavailability respectively, convey a different information. Indeed, Figure 3a,b display the appearance of almost perfect “Gaussian” distributions showing an initial phase of relatively lower biomass activity, then peaking before declining.

It is particularly interesting to observe that the period of relatively lower activity lasted about 860 h (36 days) for the bioaugmented system, while lower activity lasted for about 576 h (24 days) for the bioaugmented with enhanced bioavailability system. The peak in bioremediation activity was reached after 2117 h (88 days) for the bioaugmented system and 1149 h (48 days) for the bioaugmentation with enhanced bioavailability system. Clearly, the addition of the anionic surfactant Tween 80 seems to “speed-up” bioremediation. However, is this a significant advantage in bioremediation? Further analysis of the ratio of the cumulative CO_2_ results at the end of the experiments for the Bio_Stim and Bio_Cont systems is around 1.3 while the ratio for Bio_Aug and Bio_Avail is around 1.44. This can be seen in the combined plots in Figure 1a,b. On the other hand, the ratio of Bio_Aug and Bio_Cont is just over 75 while the ratio of Bio_Avail and Bio_Cont is around 52, as can be deduced from the scales in Figure 1a,b as appropriate. Clearly, there is very significant positive effect using bioaugmentation and enhancing bioavailability. However, adding the anionic surfactant Tween 80 merely accelerated the rate of hydrocarbon mineralization by shortening the initial “adaptation” phase and moving forward the peak activity. At the same time, the rate of bioremediation declined rapidly for the Bio_Avail system as seen in Figure 3b compared to Figure 3a representing the Bio_Aug system.

### 3.2. TPH Removal Efficiency

**Figure 4:**
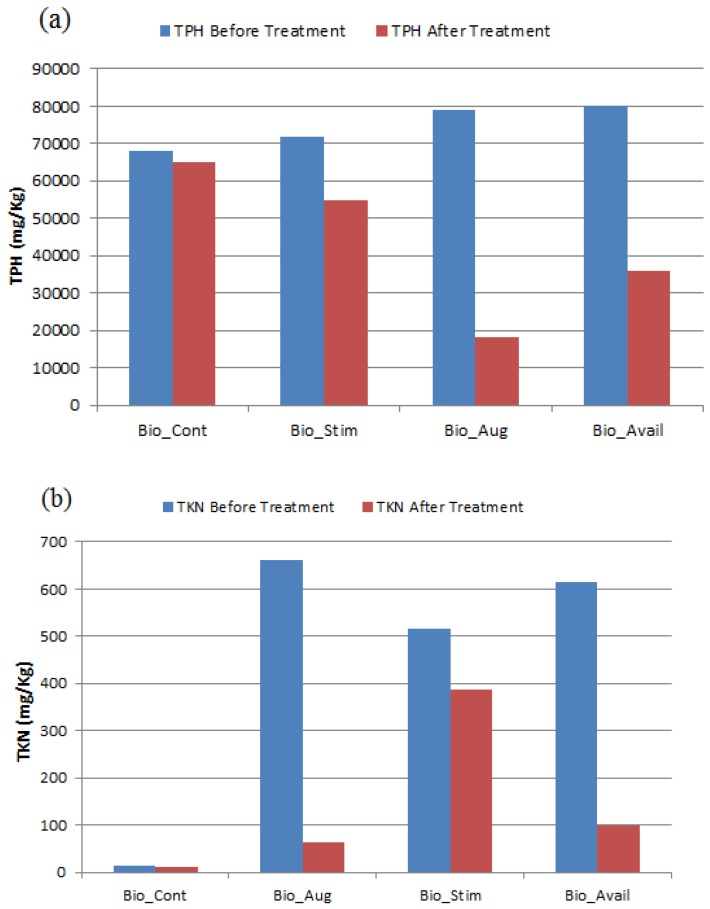
(**a**) TPH and (**b**) TKN before and after biopile treatment.

In terms of TPH reduction after biopile treatment over a period of 3720 h (155 days), one can see from Figure 4a that best results were obtained with the Bio_Aug biopile system with a reduction of just over 77% while the Bio_Avail biopile system gave a 55% TPH reduction. The Bio_Stim system gave a reduction of just over 23% and the Bio_Cont gave only a reduction of TPH of just over 4%. This trend is within expectation qualitatively. How can one explain the relatively lower performance of the Bio_Avail system compared to the Bio_Aug system? One needs to look at one of the most important nutrients depletion, namely nitrogen, shown in Figure 4b. 

### 3.3. Nitrogen Nutrient Effect

Indeed, Figure 4b indicates that the total nitrogen was depleted by just over 90% for the Bio_Aug system and by nearly 85% for the Bio_Avail system. If we assume that the rate of nitrogen depletion mirrors the bioremediation process, one can conclude that nitrogen nutrient was depleted sooner than in the case of the Bio_Aug system since the Bio_Avail system activity peaked much earlier than that of Bio_Aug. This may well have led to the faster decline in bacterial activity in the Bio_Avail system. This result is consistent with literature reporting on the effect of nitrogen deficiency [13].

### 3.4. Bioaccessibility Concept

**Figure 5:**
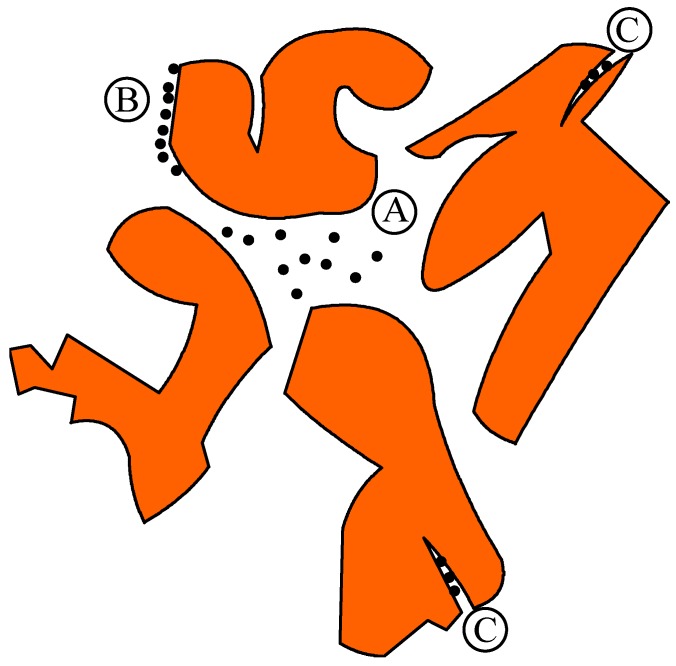
Conceptual representation of bioavailability and bio-accessibility of organic contaminants in soils.

The soil bioremediation process is quite complex and there are no simple or established models that accurately describe it. The difficulty arises from the unknown distribution of the hydrocarbon nutrient and other nutrients relative to that of the biomass within the soil matrix. Because of this complication an additional term has sometimes been added in bioremediation investigations [18], namely “bioaccessibility” alongside “bioavailability. Figure 5 is an attempt to clarify this important point which can help interpret some observations in assisted soil bioremediation by means of additives like surfactants and nutrients. In Figure 5 we have three hypothetical situations labelled as A, B and C. The dark dots indicate nutrients (hydrocarbons and other nutrients like NPK) surrounded by soil particles. The following situations can be considered:

A1: Hydrocarbons/nutrients can be “bioavailable” if bacteria are also located in the same spot;
A2: Hydrocarbons/nutrients can be “bioaccessible” if bacteria are not located in the same spot but these nutrients may be transported to the spots where bacteria are located;
B: Hydrocarbons/nutrients are adsorbed on soil particles and can be released to become either “bioavailable” or “bioaccessible” as per case A1 or A2;
C: Hydrocarbons/nutrients can be trapped and hence not “bioaccessible” to bacteria.

In practice the above possibilities exist both spatially and temporally during the bioremediation. The dynamics of these possibilities are somewhat reflected in the daily respiration rates shown in Figure 2 and Figure 3. Occasional spikes in respiration rates can be explained in terms of situation B described above where additional nutrients become available and consumed. The effect of non ionic surfactant Tween 80 can also be interpreted as “facilitation” of transport of nutrients through situation A2 above. This may well explain the earlier peaking of bioremediation activity for the Bio_Avail system.

### 3.5. Residual PAH Post Biopile Treatment

The traditional hydrocarbon contaminated soil bioremediation treatment performance is measured by means of the “bulk parameter” TPH which is convenient but does not indicate the nature of residual hydrocarbons. Because of the potential health risks associated with residual hydrocarbons, a certain category of heavy hydrocarbons deserve particular attention, namely polyaromatics. 

The polyaromatic hydrocarbons (PAHs) analyzed at the end of the biopile treatment processes are shown in Table 2 as residual PAH indicate that there is no obvious pattern of prevalence with respect to the biopile system used. This suggests that amongst the complex mixture of hydrocarbons in crude oil, PAH’s are probably last to be mineralized and would most likely require better adapted biomass. The nitrogen depletion in this work probably prevented further TPH reduction that may result in less PAH residuals.

**Table 2:** Residual polyaromatics (PAH, in mg/kg) after treatment.

PAH	Bio_Cont	Bio_Aug	Bio_Stim	Bio_Avail
Naphthalene	2.65	0.54	0.70	0.49
Acenaphthylene	ND *	0.09	0.16	0.10
Acenaphthene	0.38	0.31	0.42	0.33
Flourene	3.87	2.88	4.14	3.30
Phenanthrene	10.30	7.48	11.0	0.21
Anthracene	0.04	0.03	0.05	0.03
Fluoranthene	11.30	9.55	14.9	9.78
Pyrene	2.64	2.13	3.23	2.30
Benzo(a)anthracene	1.10	0.65	0.96	0.72
Chrycene	4.87	3.43	5.22	3.83
Benzo(b)flouranthene	0.05	0.09	0.03	0.08
Benzo(k)flouranthene	0.07	0.20	0.38	0.07
Benzo(a)pyrene	0.20	0.05	0.20	0.05
Dibenzo(a,h)anthracene	0.05	0.08	0.19	0.04
Benzo(g,h,i)perylene	0.28	0.12	0.26	0.14
Indeno(1,2,3-cd)pyrene	0.12	0.18	0.14	0.10

ND * means not detected.

## 4. Conclusions

Bioaugmentation with biostimulation of desert soil gave the best overall result in terms of TPH reduction with a 77% reduction over a period of 156 days. An effect of surfactant Tween 80 was observed in terms of an accelerated bioremediation process with a peak respiration activity after 48 days compared to 88 days for the “bioaugmented” system. The accelerated “bioavailable” system respiration declined sooner than the “bioaugmented” system through rapid depletion of nitrogen nutrient and gave a 55% TPH reduction over 156 days. The residual hydrocarbon in all four biopiles contained PAHs in quantities that may be considered as hazardous. 

## Appendix

**Table A1:** Sahel oil field clean soil properties.

Texture	Average Particle Size (μm)	Bulk Density (g/L)	pH	Conductivity (μS cm^-1^)	Field Capacity % (wt/wt)	Oil Absorption Capacity % (wt/wt)
Sandy loam (86.52% sand, 13.48% silt, 0% clay)	150	1.6	7.81	118	23	17
